# Common mental disorders among medical students: systematic review and meta-analysis of Brazilian studies

**DOI:** 10.1590/1516-3180.2021.0851.R1.27012022

**Published:** 2022-08-08

**Authors:** Silvio José Batista Soares, Cláudia Fernanda Garcez Fernandes, Renata Tabalipa, Felipe Kogima, Marcelo Augusto Moreira Jubini, Isabella Martins Vieira Dias, Victor Emanuel Miranda Soares, Severina Silva Amaral, Michele Santos da Cruz, Paulo Henrique Guerra

**Affiliations:** I Undergraduate Medicine Student, Department of Medicine, Universidade Federal da Fronteira Sul (UFFS), Chapecó (SC), Brazil.; II Undergraduate Medicine Student, Department of Medicine, Universidade Federal da Fronteira Sul (UFFS), Chapecó (SC), Brazil.; III Undergraduate Medicine Student, Department of Medicine, Universidade Federal da Fronteira Sul (UFFS), Chapecó (SC), Brazil.; IV Undergraduate Medicine Student, Department of Medicine, Universidade Federal da Fronteira Sul (UFFS), Chapecó (SC), Brazil.; V Undergraduate Medicine Student, Department of Medicine, Universidade Federal da Fronteira Sul (UFFS), Chapecó (SC), Brazil.; VI Undergraduate Medicine Student, Department of Medicine, Universidade Federal da Fronteira Sul (UFFS), Chapecó (SC), Brazil.; VII Undergraduate Medicine Student, Department of Medicine, Universidade Federal da Fronteira Sul (UFFS), Chapecó (SC), Brazil.; VIII Undergraduate Medicine Student, Department of Medicine, Universidade Federal da Fronteira Sul (UFFS), Chapecó (SC), Brazil.; IX MSc. Doctoral Student, Postgraduate Program on Nutrition, Universidade de São Paulo (USP), São Paulo (SP), Brazil.; X DSc. Associate Professor, Department of Medicine, Universidade Federal da Fronteira Sul (UFFS), Chapecó (SC), Brazil.

**Keywords:** Mental health, Education, medical, undergraduate, Review [publication type], Brazil, Common mental disorders, Prevalence, Medical students

## Abstract

**BACKGROUND:**

Common mental disorders (CMDs) have been correlated with consequences in different domains of life.

**OBJECTIVE:**

To summarize the prevalence rates of CMDs and factors associated with them among students at Brazilian medical schools.

**DESIGN AND SETTING:**

Systematic review and meta-analysis of studies developed in Brazilian medical schools.

**METHODS:**

In October 2021, searches were carried out in seven electronic databases, in Google Scholar and in reference lists. Observational studies reporting prevalence rates of CMDs among students at Brazilian medical schools were sought. Variables associated with CMDs arising from multivariate regression models were included in the synthesis. A meta-analysis was developed using a random-effects model and the risk of bias was assessed using an instrument developed from previous references.

**RESULTS:**

Fourteen original studies were included. The pooled prevalence rate of CMDs among undergraduate students at Brazilian medical schools was 43.3% (95% confidence interval = 38.9% to 47.6%; I^2^ = 87%; n = 3,927). Among the nine studies in which multivariate analyses were conducted, five showed risk associations between CMDs and medical school-related dissatisfactions, among which the desire to abandon the medical course can be highlighted (n = 3). In three studies, CMDs were associated with sleep indicators.

**CONCLUSION:**

Considering that the prevalence of CMDs among medical students is higher than in the general population, we recommend that Brazilian medical schools should give greater attention to this topic, and should enable expansion of care offerings relating to mental health.

**SYSTEMATIC REVIEW REGISTRATION:**

Prospective Register of Systematic Reviews (PROSPERO) database (CRD42020142184).

## INTRODUCTION

Medical courses are, as a rule, characterized by an integral routine of theoretical and practical activities, early insertion in a *praxis* that has little margin for errors and permanent contact with illness and death. In the Brazilian context, it is recognized that these demands are associated with different health risk behaviors, such deprivation of sleep^1^ and leisure,^2^ which, in turn, are determinants of mental health.^3,4^

Since the mental health indicators observed among Brazilian medical students are generally poorer than those observed in the general population,^5^ the mental health of medical undergraduate students is an emerging agenda in Brazilian research, enhanced by recognition of the most drastic outcomes associated with it.^3^ For example, common mental disorders (CMDs) are one of the mental health indicators that have been studied in these populations.^5^

CMDs present as a mixture of somatic, anxiety-related and depressive symptoms, such as insomnia, irritability, forgetfulness and difficulty in concentrating, among others.^6^ However, these symptoms do not meet sufficient formal criteria to be diagnosed as depression or anxiety, according to the classifications of the DSM-V (Diagnostic and Statistical Manual of Mental Disorders, 5^th^ edition) and the International Classification of Diseases, 11^th^ revision (ICD-11).^7^

Nonetheless, the literature available suggests that CMD symptoms among Brazilian medical students are already observed in the early stages of the medical course.^8^ In this sense, it can be understood that early recognition of CMD prevalence rates, as well as the factors associated with these disorders, can provide support for efforts to address them at different levels. These can range from the strategies available for mental healthcare in medical schools to a broader view of the curricula of Brazilian medical schools.

Considering the distinct biopsychosocial impairments associated with CMDs, identifying the prevalence rates of CMDs and factors associated with them can support development of preventive strategies involving these populations.

## OBJECTIVE

Thus, the aim of this study was to identify and statistically summarize CMD prevalence rates and factors associated with these disorders among students at Brazilian medical schools.

## METHODS

We developed a systematic review of the literature, with meta-analysis. Its protocol was previously registered in the International Prospective Register of Systematic Reviews (PROSPERO) database (CRD42020142184) and its design and report were developed from the items of the Preferred Reporting Items for Systematic Reviews and Meta-Analyses (PRISMA) checklist.^9^

Our inclusion criteria were formulated from the possible items of the “PICOS” structure, considering the following: (I) Population: students at Brazilian medical schools, without restrictions regarding the stage of the course and the profile of the university (i.e. public or private); (II) Outcome: “common mental disorders”, not considering studies that addressed other terminologies and other specific psychiatric disorders/diseases, such as anxiety, stress and depression; and (III) Study design: observational studies (e.g. cross-sectional or cohort studies), without restriction as to their representativeness (i.e. whether local, regional or national), reported in English, Portuguese or Spanish. It is worth mentioning that, in accordance with the study designs of interest, the PICOS items “Intervention” and “Control” were not applicable.

On the other hand, dissertations, theses, abstracts and preprints were not considered for the synthesis. Nor was research involving students on healthcare courses in which no stratified analysis of CMD prevalence rates among medical students was presented.

As our secondary outcome, we sought to identify the variables associated with CMDs in studies in which multivariate regression model-based analyses were conducted (i.e. independent of the regression type and effect measurements presented). This was done while considering their robustness in adjustment of confounders.

Potential studies were screened up to the cutoff date of October 4, 2021, using three strategies: (I) application of search strategies in seven electronic databases (Embase, Lilacs, PsycINFO, PubMed, SciELO, Scopus and Web of Science), based on the syntaxis developed for PubMed: ((((common mental disorder [Text Word]) or (common mental disorder [Text Word])) and (((medicine [Text Word]) or (medical school [Text Word])) or (medical schools [Text Word]))) and ((Brazil [Text Word]) or (Brazilian [Text Word])); (II) searches through the first 200 records of Google Scholar, using the terms “common mental disorder(s)”, “medicine”, “medical” and “Brazil”; and (III) manual searches in the reference lists of articles that were evaluated through their full texts. In the Lilacs and SciELO databases and in Google Scholar, the terms were also searched in Portuguese. There were no restrictions regarding the year of publication of the articles.

The operational process, which involved analysis of titles, abstracts and full texts, was conducted by four independent researchers, with collaboration from two other researchers to resolve doubts and establish consensus. Searches in Google Scholar were conducted by two researchers, also independently. Data extraction, performed in an electronic spreadsheet, was divided into descriptive information, methods and results, and was also conducted by two researchers, independently, with subsequent verification of the data conducted by another six members, organized in pairs. The descriptive synthesis was elaborated from the main topics of the data extraction worksheet.

The risk of bias of the studies included was assessed considering our primary outcome (i.e. summarized CMD prevalence rates). For this, an adapted version of the quality assessment tool for quantitative studies of the Effective Public Health Practice Project (EPHPP)^10^ was used. This covers the following five methodological domains: (I) Selection bias (i.e. information about the sample, such that studies involving all phases of the medical course were considered to present “low risk of bias”); (II) Study design (i.e. methods used in sampling); (III) CMD assessment tool (e.g. which tool was used, a report on its previous validation and information that enables replication of the measurement, in the case of questionnaires developed specifically for the study); (IV) Losses and dropouts (i.e. information on losses and dropouts, along with the percentage of students whose data were analyzed, compared with the initially proposed number); and (V) Analysis protocol (e.g. analysis plan and adequacy of the method used to identify the prevalence of CMDs in the sample). This instrument may be requested from the corresponding author.

A random-effects meta-analysis was conducted, based on the original prevalence rate data from each individual study and the respective 95% confidence interval (CI). Given that variability data is not reported with the 95% CI in many studies, these data were manually calculated from the sample size and the prevalence of identified CMDs and subsequently checked in the statistical software.

To conduct the analysis, the Review Manager software was used (version 5.4; Cochrane Collaboration, 2020). Thus, the summarized effect was constructed from the random model, considering the differences between the samples (e.g. phase/year of the course and type of institution). The I^2^ statistic was used to assess heterogeneity between studies: this was classified as “moderate heterogeneity” when the summarized effects using I^2^ were between 50% and 74%, or as “high heterogeneity” when I^2^ ≥ 75%, as suggested in the study by Higgins et al.^11^

## RESULTS

**Figure 1:**
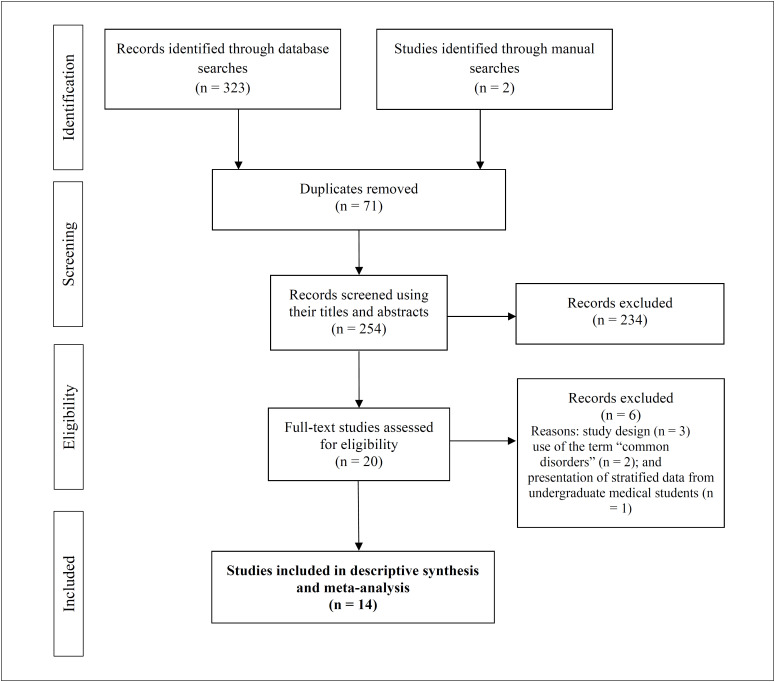
Flowchart of systematic review and meta-analysis.

Overall, the searches retrieved 325 potential studies (Figure 1). After identification and removal of duplicates (n = 71), 254 studies remained for assessment using their titles and abstracts. At the end of this stage, 20 studies were considered eligible for evaluation using their full texts. Six of these were subsequently excluded, for the following reasons: study design (n = 3); no use of the term “common mental disorders” (n = 2); and lack of presentation of stratified data from undergraduate medical students (n = 1). Thus, the synthesis was developed based on the data from 14 original studies.^8,12–24^

Regarding geographical location, the studies were conducted in ten cities (Aracajú, Blumenau, Botucatu, Jequié, Montes Claros, Ponta Grossa, Salvador, São José do Rio Preto, Vila Velha and Vitória), located in seven Brazilian states (Bahia, Espírito Santo, Minas Gerais, Paraná, Santa Catarina, Sergipe and São Paulo). Regarding study design, twelve studies were cross-sectional^11–15,17–24^ and two were longitudinal.^8,16^ The samples ranged from 40^16^ to 477^20^ undergraduate medical students, with a higher frequency of women in nine of these studies (64.3%). Eight studies involved students enrolled in all stages of the medicine course (57.1%) (Table 1).^13,16,17–20,22,24^

**Table 1:** Descriptive characteristics of the studies included (n = 15)

References	Study location (year of data collection)	Number of participants (% of women in the sample)	Stages of medical course covered	Tool used for CMD assessment	Risk-of-bias domains				
					Selection bias	Study design	CMD assessment tool	Losses and dropouts	Statistical analysis protocol
Almeida et al.^12^	Salvador-BA (2005)	223 (48)	2, 4, 6 and 8	SRQ-20	Moderate	Weak	Strong	Strong	Strong
Aragão et al.^13^	nd (nd)	428 (66)	1-12	GHQ-12	Strong	Moderate	Strong	Moderate	Strong
Bellinati et al.^14^	São José do Rio Preto-SP (nd)	118 (77)	1, 5 and 8	SRQ-20	Moderate	Strong	Strong	Strong	Strong
Costa et al.^15^	Aracajú-SE (2006)	473 (50)	2-12	SRQ-20	Strong	Strong	Strong	Strong	Strong
Costa et al.^16^	Aracajú-SE (2006)^a^	40 (58)	1-12^b^	SRQ-20	Strong	Strong	Strong	Strong	Strong
Ferreira et al.^8^	Ponta Grossa-PR (2013)^a^	134 (48)	1-8	SRQ-20	Moderate	Strong	Strong	Strong	Strong
Fiorotti et al.^17^	Vitória-ES (2007)	229 (50)	1-12	SRQ-20	Strong	Weak	Strong	Strong	Strong
Grether et al.^18^	Blumenau-SC (2017)	340 (67)	1-12	SRQ-20	Strong	Weak	Strong	Moderate	Strong
Lima et al.^19^	Botucatu-SP (2002)	455 (61)	1-12	SRQ-20	Strong	Strong	Strong	Strong	Strong
Lima et al.^20^	Botucatu-SP (2011)	477 (59)	1-12	SRQ-20	Strong	Weak	Strong	nd	Strong
Medeiros et al.^21^	Montes Claros-MG (2015)	101 (64)	1	GHQ-12	Weak	Weak	Strong	Moderate	Strong
Melado et al.^22^	Vila Velha-ES (2018)	360 (60)	1-12	SRQ-20	Strong	Strong	Strong	Strong	Strong
Santos et al.^23^	Jequié-BA (2016)	115 (47)	nd	SRQ-20	nd	Weak	Strong	Weak	Strong
Silva et al.^24^	Botucatu-SP (nd)	434 (58)	1-12	SRQ-20	Strong	Strong	Strong	Strong	Strong

a Study with repeated measurements in the same sample

b A group of students who were followed throughout the six years of their medical course; BA = Bahia; ES = Espírito Santo; GHQ-12 = General Health Questionnaire; MG = Minas Gerais; nd = not described; PR = Paraná; SC = Santa Catarina; SE = Sergipe; SP = São Paulo; SRQ-20 = Self Reporting Questionnaire.

Two questionnaires for CMD assessment were identified: the Self Reporting Questionnaire (SRQ-20), in 12 studies (80%);^8,12,14–20,22–24^ and the General Health Questionnaire (GHQ-12), in the other two studies.^13,21^ The risk-of-bias ratings were low in the majority of the methodological domains assessed. Specifically regarding the “statistical analysis” and the “CMD assessment tool”, all the studies included were assessed as presenting low risk of bias. On the other hand, due to limitations in the sampling processes (i.e. convenience-based samples), the “study design” domain was the one most impacted by studies classified as having high risk of bias (n = 6) (Table 1).^12,17–19,21,23^

**Figure 2:**
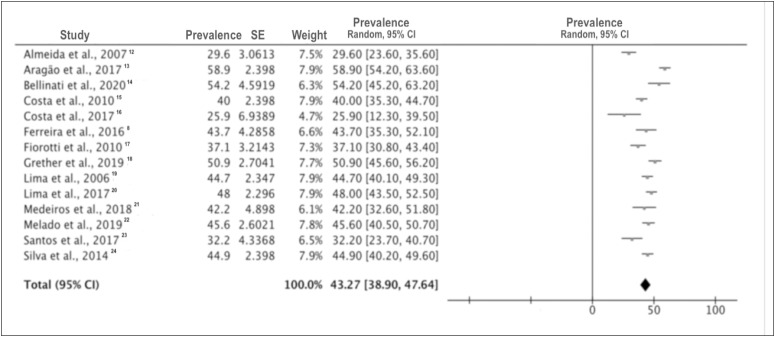
Meta-analysis on the prevalence of common mental disorders among Brazilian medical school students.

The pooled prevalence rate for CMDs was 43.3% (95% CI = 38.9% to 47.6%; I^2^ = 87%; n = 3,927) among the undergraduate medical students at Brazilian medical schools (Figure 2). The highest and lowest CMD prevalence rates were found in the studies by Costa et al. (25.9%) and Grether et al. (50.9%), respectively. In view of the high heterogeneity in the primary analysis, a subgroup analysis on the studies that covered undergraduate students from all medicine course stages was conducted. The pooled prevalence was 45.7% (95% CI = 40.8% to 50.7%; I^2^ = 85%) (data not shown).

**Figure 3:**
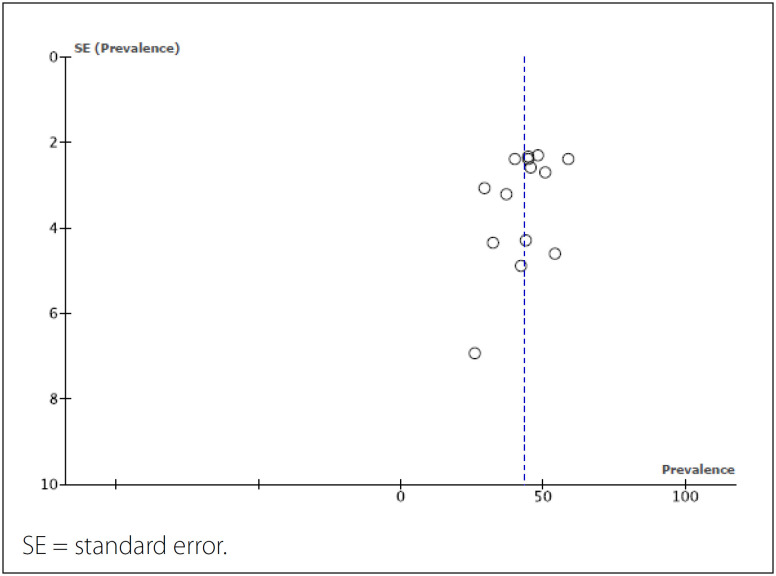
Funnel plot from the meta-analysis on the prevalence of common mental disorders among Brazilian medical school students

A funnel plot for the overall pooled prevalence rate of CMDs is presented in Figure 3. From the funnel shape, it was assessed that there was no significant publication bias in this meta-analysis.

Multivariate regression models to identify factors associated with CMDs were developed in nine studies (64.3%). Despite the differences relating to the types of regression and variables used for adjustment, the results suggest that CMDs were frequently associated with dissatisfaction relating to the medicine course.^12,15,19,22,24^ The factors of “desire to quit the course”^19,22,24^ and “feeling uncomfortable with the course”^12,15^ can be highlighted. Furthermore, risk associations between CMDs and sleep indicators^8,12,22^ were also shown (Table 2).

**Table 2:** Factors associated with common mental disorders that were identified in studies in which multivariate regression analyses were conducted (n = 9)

References	Variables associated with common mental disorders
Almeida et al.^12^	Sleep pattern changes (OR = 6.7; 95% CI = 3.2 to 13.8) Not having own transportation (OR = 3.9; 95% CI = 1.9 to 8.0) Did not have a job (OR = 3.5; 95% CI = 1.0 to 12.4) Not exercising (OR = 2.2; 95% CI = 1.0 to 4.5)
Costa et al.^15^	Not believing in acquiring skills to become a good doctor (OR = 2.8) Being uncomfortable with course activities (OR = 3.8) Considering oneself emotionally tense (OR = 2.1) Not considering oneself happy (OR = 2.9) Finding that the course was less than expected (OR = 1.6) Having a previous diagnosis of mental disorder made by a psychiatrist (OR = 3.8)
Costa et al.^16^	Factors associated in analysis carried out in the 5^th^ year of the course: Female (PR = 1.4) Being from state capital (PR = 1.9) Finding that the course was less than expected (PR = 3.2) Being uncomfortable with course activities (PR = 2.1) Being dissatisfied with teaching strategies (PR = 1.4) Feeling that the course was not a source of pleasure (PR = 2.1)
Ferreira et al.^8^	At beginning of the semester: Monthly family income per capita ≤ R$ 2,000.00 (OR = 3.2; 95% CI = 1.3 to 8.0) Poor sleep quality (OR = 3.3; 95% CI = 1.1 to 9.5) At end of the semester: Poor sleep quality (OR = 3.3; 95% CI = 1.2 to 7.9)
Fiorotti et al.^17^	Not receiving the emotional support needed (OR = 7.4; 95% CI = 3.1 to 17.9) Report of “difficulty in answering questions in the classroom due to shyness” during childhood or adolescence (OR = 2.5; 95% CI = 1.0 to 6.1)
Lima et al.,^19^	Difficulty in making friends (OR = 2.0; 95% CI = 1.2 to 3.3) Poor evaluation of school performance (OR = 1.7; 95% CI = 1.1 to 2.7) Thinking about quitting the course (OR = 5.0; 95% CI = 1.1 to 2.7) Not receiving the emotional support needed (OR = 4.6; 95% CI = 2.0-9.9)
Melado et al.^22^	Mental disorder in the family (RR = 1.2; 95% CI = 1.0 to 1.5) Poor sleep quality (RR = 1.5; 95% CI = 1.2 to 1.9) Fear that affected school performance (RR = 1.3; 95% CI = 1.0 to 1.8) Feeling rejected by friends (RR = 1.5; 95% CI = 1.1 to 1.9) Thinking about quitting the course (RR = 1.7; 95% CI = 1.3 to 2.2) Physical discomfort during the test (RR = 1.6; 95% CI = 1.2 to 2.2)
Santos et al.^23^	Physical domain (adjusted β = 0.9; 95% CI = 0.89 to 0.97) Psychological domain (adjusted β = 0.9; 95% CI = 0.91 to 0.99)
Silva et al.^24^	Feeling rejected in the last year (OR = 2.5; 95% CI = 1.5 to 4.2) Having thought about or thinking about quitting the course (OR = 6.9; 95% CI = 2.4 to 19.4) Interaction (OR = 2.4; 95% CI = 1.4 to 4.2)

CI = confidence interval; OR = odds ratio; PR = prevalence ratio; RR = relative risk; β = beta; R$ = Real, the national currency of Brazil, also known as Brazilian Real (BRL).

## DISCUSSION

Based on the data from 14 original studies, the pooled prevalence rate of CMDs was 43.3% among undergraduate students at Brazilian medical schools. Compared with a previous study,^5^ which reported a pooled prevalence of 31%, our study had two specificities: inclusion of studies that specifically used the ‘common mental disorders’ terminology and inclusion of studies that assessed CMDs through questionnaires other than the SRQ-20.

Specifically regarding CMD assessment, it is important to highlight that the GHQ-12 is classified by the Brazilian Federal Council of Psychology as an “unfavorable psychological test” and, therefore, is not indicated for psychologists’ professional activities.^25^ However, these studies using the GHQ-12 were kept in our synthesis,^13,21^ for two reasons: our recognition of a Brazilian validation study on the GHQ-12 in which the SRQ-20 was used as a comparison;^26^ and our recognition that the GHQ-12 has been used in other population-based studies.^27,28^

Even though no nationwide survey exists, our findings corroborate the understanding that the prevalence of CMDs observed among Brazilian undergraduate medical students is higher than that of the general adult population.^7,29^ We believe that this result is important and can contribute to efforts relating to the debate about the structure of medical courses offered in Brazil, through highlighting the need for provision of specialized mental healthcare for undergraduate students.

From this perspective, one of the actions can consist of monitoring of mental health indicators (e.g. CMDs, burnout syndrome, stress and anxiety) from the outset of the medical course. Previous research has suggested that high levels of stress are observed in pre-university preparatory courses,^30^ and absence of care may lead to maintenance and/or worsening of risk indicators as the undergraduate course progresses. Among the studies included in the synthesis, only one specifically involved a sample of freshmen.^21^ In that study, in addition to the high prevalence of CMDs (i.e. 42.2%), presence of other negative health indicators was suggested, such as pathological levels of daytime sleepiness, depressive symptoms of varying degrees, emotional exhaustion and depersonalization.^21^

Identifying these indicators in the early phases of the medicine course can be of interest, in order to avoid chronicity among them, along with the more deleterious outcomes mentioned above.^3^ Another suggestion is to investigate whether there are phases, course cycles (i.e. basic, clinical or internship) or even moments in each semester when the risk of developing CMDs is higher. It is worth mentioning that in the longitudinal studies by Ferreira et al.^8^ and Costa et al.,^16^ it was suggested that the prevalence of CMDs increases over the semester^8^ and over the years,^16^ respectively.

As our secondary result, we also showed that course-related dissatisfaction^12,15,19,22,24^ and sleep indicators^19,22,24^ were associated with CMDs. Even though three studies pointed out that CMDs were associated with the desire to drop out of medical school, it has been recognized in the literature available that medicine courses have lower dropout rates than nursing, pharmacy and dentistry courses.^31^ This finding can largely be explained by different perspectives, such as remuneration, job expectations and social recognition.^32^ Nonetheless, apart from the social role of medical doctors and the social constructs that permeate the profession, these associations relating to dissatisfaction and frustration with the course suggest that there is a need for periodic assessment of course workloads. Adoption of innovative strategies that go beyond purely technicist and poorly humanized approaches can also be highlighted.^33^

Regarding sleep indicators, it has been recognized that sleep disorders are associated with other mental health indicators, such as anxiety and depression.^34^ The routines required by different curricular components and the pressure for better performance in tasks can lead to constant sleep deprivation. In addition, there is also high and recurrent consumption of psychostimulant substances (e.g. energy drinks and caffeine) among medical students, to prolong their state of alertness.^35^ Thus, beyond guidance about the harm of sleep deprivation, better care in the internal organization of courses is suggested, so that overlapping of activities, tests and/or important tasks on specific dates or in specific weeks can be avoided.

In addition, since most of the studies included here had cross-sectional designs, we would recommend that longitudinal studies should be conducted. These would not only investigate whether there are phases/cycles of higher risk of CMDs during the medical course, but also provide understanding of the possible causal relationships between the variables. Therefore, we would emphasize that caution is required in interpreting the findings from this study, since most of the studies included were cross-sectional. This formed a limitation on deeper discussion of temporality and causality, i.e. whether CMDs are the cause or the consequence of sleep disorders.

## CONCLUSION

Our study showed that the pooled prevalence rate of CMDs was 43.3% among the undergraduate medical students. It also showed that risk associations existed between CMDs and course-related dissatisfaction and sleep indicators. Considering that the prevalence of CMDs among medical students is higher than in the general population, we recommend that Brazilian medical schools should give greater attention to this topic and should enable expansion of care provision relating to mental health.
